# Rising Pediatric Morbidity and Mortality in the Developing World

**DOI:** 10.7759/cureus.14728

**Published:** 2021-04-28

**Authors:** Haider Ali, Sina Aziz

**Affiliations:** 1 Pediatrics, Karachi Medical & Dental College, Karachi, PAK

**Keywords:** pediatrics, morbidity, mortality, infectious diseases, epidemiology, immunization, pneumonia, malaria, meningitis, diarrheal diseases

## Abstract

Pediatric morbidity and mortality are on the rise in certain regions of the world specifically the low-income countries where no goals are being met in this regard. In comparison to the developed countries, the rate of decline in morbidity and mortality is dawdling. Disease trends show that there is a resurgence of diseases that are already major contributors to this healthcare crisis (big players like pneumonia, malaria, and meningitis), whereas no significant decrease is being noted in the others. Seasonal and cyclical trends along with other factors such as antibiotic resistance and deficient vaccination coverage in the low socioeconomic class (due to decreased availability or lack of awareness) can be counted as major precipitating factors and policies should be aimed towards rectification.

## Introduction

Trends in morbidity and mortality in the pediatric age group are good indicators of the status of health in developing countries. These indicators highlight the main precipitating factors which cause the increase in morbidity and mortality and help in determining goals and allocating resources to areas of healthcare that need to be addressed with the utmost importance. According to the World Health Organization (WHO), approximately 5.2 million children under the age of five died globally in 2019, which means greater than 14,000 under-five deaths occurred per day [1]. Low-income countries, where the under-five mortality rate is 15 times higher than in high-income countries, make a significant contribution to this figure [2]. Studied in-depth, morbidity and mortality rates are vital for a thriving society and highlight the dynamics of precipitating factors. But are these factors being focused on by policymakers in the developing world? And if so, why is there still no substantial decrease in pediatric morbidity and mortality?

Neonatal deaths dropped worldwide from five million in the year 1990 to 2.4 million in 2019, while the under-five mortality rate diminished 58% from 93 deaths per 1000 live births in 1990 to 37.7 per 1000 children in 2019 [2]. In the age group of five to nine years, the probability of dying decreased globally from 9.8 per 1000 children in 1990 to 3.8 per 1000 children in 2019 which is a significant drop of more than half the number of children. However, in Pakistan this value has seen no substantial descent over the years, reducing from 8.1 per 1000 children in 1990 to 4.6 per 1000 children in 2019 (being almost completely stagnant between 2003 and 2011 at a value of 5.6). The under-five mortality rate is at 67.2 deaths per 1000 children in 2019, reducing only 51.8% from 138.8 per 1000 in 1990, earning Pakistan the third position in the top 10 countries with the highest number of deaths for children under five years [1,3]. All these numbers have contributed to United Nations Children's Fund (UNICEF) declaring Pakistan the riskiest place to be born with a newborn mortality rate of 45.6 deaths per 1000 live births and newborns having a one in 22 chances of death [4].

The developing countries are disproportionately burdened with under-five mortality, with 80% of deaths occurring in sub-Saharan Africa and Southern Asia alone. India, Nigeria, Pakistan, the Democratic Republic of the Congo, Ethiopia, and China together account for half of these figures, and hence, require internationally effective measures to be taken [5].

After the success of the Millennium Development Goals (MDGs), the United Nations came up with the Sustainable Development Goals (SDGs) to build on the momentum gathered by MDGs and work on the unachieved targets. These goals were specifically focused on the poor and developing countries to reduce morbidity and mortality and improve the quality of health and life in these areas. The fourth goal of the MDGs was to reduce child mortality by 2015, which can be considered as a success since global under-five mortality dropped by more than half, from 90 to 43 deaths per 1000 live births, between 1990 and 2015 [6]. The story in Pakistan was different, however, with the under-five mortality rate at 76.1 deaths per 1000 in 2015, whereas the target being 52 deaths per 1000 [7]. Hence, the SDGs were developed to continue the progress, and goals were set for the year 2030, among which goal no. three is to ensure healthy lives and promote well-being for all ages [8].

In the under-five population around the world, the leading causes of mortality are preterm birth complication, pneumonia, birth asphyxia, diarrhea, and malaria, and more than 50% of these deaths are avertible by simple and affordable interventions. Approximately 45% of these deaths are due to nutrition-related factors. Comparatively, in Pakistan, the leading causes of death are prematurity, birth asphyxia and trauma, sepsis, acute respiratory infections, and congenital anomalies [9].

## Materials and methods

Since 2013, we started documenting each child's disease and its outcomes in the pediatric ward and pediatric intensive care unit (PICU) of a tertiary care hospital in Karachi, Pakistan. We made an observation that despite immunization being freely available in the catchment area of the hospital, and all over Pakistan via Expanded Program on Immunization (EPI), a large number of children were still suffering from EPI-covered diseases. Among the various reason for low immunization coverage, one important reason is the lack of knowledge and awareness about immunization and a mindset that considers vaccines to be harmful towards child development.

Due to the concern of increasing admission rates of infectious diseases, along with other preventable ailments, we decided to present our views regarding the morbidity and mortality of diseases seen in the tertiary care hospital from 2013 to 2017 covered by EPI, and other disease patterns such as malaria, typhoid, meningitis, encephalitis, protein-calorie malnourishment (PCM), acute gastroenteritis (AGE), failure to thrive (FTT), congenital heart disease (CHD), sepsis and neonatal causes of admission (meconium aspiration syndrome and birth asphyxia).

Data were collected from a total of 3110 patients every month from 2013 to 2017 from the monthly morbidity and mortality meetings conducted in Pediatric Unit 2. Patient data, including age, gender, laboratory reports, diagnosis, vaccination status, treatment, and outcome, was discussed and documented. A final datasheet was prepared each month by the team and finalized by the senior consultant.

All the patients admitted to Pediatric Unit 2 and the PICU at the tertiary care hospital were included in the study. Since the department did not have a neonatal intensive care unit, emergency admissions of neonatology were admitted in the PICU, which has two incubators, along with a ventilator for babies weighing less than 10 kg. Hence, we have included neonatal causes too. Patients presenting to the emergency and outpatient department were excluded. A detailed history was obtained from the patients by the doctors and clinical examination was done to obtain a provisional diagnosis. Laboratory reports were requested if required, and a final diagnosis was obtained and noted. After providing the necessary treatment for the disease, the patient’s outcome (discharged/discharged on request/referred/left against medical advice/expired) was also recorded.

## Results

A total of 3110 patients presented to the tertiary care hospital during the study period of five years. Of these 3110 patients, 1779 (57.2%) were males while 1331 (42.8%) were females, hence giving us a male-to-female ratio of 1.3:1 (Table 1). Hospital admissions decreased from 2013 to 2015, with a total of 751 admissions in 2013, 639 in 2014 and 427 in 2015, and then increased steeply in the succeeding years, with 539 admissions in 2016 and 754 admission in 2017, which was the highest number of patients admitted in a year.

**Table 1:** Age, sex, vaccination status, and outcome associated distribution of pediatric patients *Newborn (0 to <1 month), infant (≥1 month and <1 year), toddler (≥1 year and <3 years), pre-school children (≥3 years and <6 years), school-going children (≥6 years and <12 years) and adolescent (≥12 years and <18 years). DOR: discharged on request; LAMA: left against medical advice

Year		2013	2014	2015	2016	2017	Total
Age	Newborn*	5 (0.7%)	21 (3.3%)	37 (8.6%)	16 (3.0%)	38 (5.0%)	117 (3.8%)
	Infant*	279 (37.1%)	217 (34.0%)	166 (38.9%)	197 (36.5%)	329 (43.6%)	1188 (38.2%)
	Toddler*	187 (24.9%)	179 (28.0%)	84 (19.7%)	134 (24.9%)	160 (21.2%)	744 (23.9%)
	Preschool*	102 (13.6%)	96 (15.0%)	55 (12.9%)	100 (18.6%)	106 (14.1%)	459 (14.7%)
	School-going*	151 (20.1%)	104 (16.3%)	67 (15.7%)	81 (15.0%)	109 (14.5%)	512 (16.5%)
	Adolescent*	27 (3.6%)	22 (3.4%)	18 (4.2%)	11 (2.0%)	12 (1.6%)	90 (2.9%)
Sex	Male	413 (55.0%)	365 (57.1%)	244 (57.1%)	328 (60.9%)	429 (56.9%)	1779 (57.2%)
	Female	338 (45.0%)	274 (42.9%)	183 (42.9%)	211 (39.1%)	325 (43.1%)	1331 (42.8%)
Vaccination Status	Complete	450 (59.9%)	400 (62.6%)	262 (61.4%)	314 (58.3%)	478 (63.4%)	1904 (61.2%)
	Partial	6 (0.8%)	59 (9.2%)	53 (12.4%)	104 (19.3%)	119 (15.8%)	341 (11.0%)
	Unvaccinated	295 (39.3%)	180 (28.2%)	112 (26.2%)	121 (22.4%)	157 (20.8%)	865 (27.8%)
Outcome	Discharged	531 (70.7%)	409 (64.0%)	272 (63.7%)	360 (66.8%)	497 (65.9%)	2069 (66.5%)
	DOR	75 (10.0%)	75 (11.7%)	29 (6.8%)	45 (8.3%)	28 (3.7%)	252 (8.1%)
	Referred	41 (5.5%)	65 (10.2%)	17 (4.0%)	15 (2.8%)	22 (2.9%)	160 (5.2%)
	LAMA	95 (12.6%)	88 (13.8%)	38 (8.9%)	94 (17.5%)	131 (17.4%)	446 (14.3%)
	Expired	9 (1.2%)	2 (0.3%)	71 (16.6%)	25 (4.6%)	76 (10.1%)	183 (5.9%)
Total		751	639	427	539	754	3110

Distributed according to age groups, infants (≥1 month and <1 year) occupied the largest part of the pediatric population visiting the hospital; 1188 (38.2%), followed by toddlers (≥1 year and <3 years); 744 (23.9%), school-going children (≥6 years and <12 years); 512 (16.5%), pre-school children (≥3 years and <6 years); 459 (14.7%), newborns (0 to <1 month); 117 (3.8%) and adolescents (≥12 years and <18 years); 90 (2.9%).

Most of the patients admitted to the pediatric unit and pediatric intensive care unit were discharged after treatment. Of the 3110 patients, a total of 2069 (66.5%) patients were treated successfully and discharged by the tertiary care center. Out of the rest of the patients, 252 (8.1%) were discharged on request, 160 (5.2%) were referred to other hospitals, and 446 (14.3%) were left against medical advice (LAMA). In the study period, 183 children expired under the care of the tertiary care hospital, which gives us a mortality rate of 58.8 per 1000 children.

**Figure 1:**
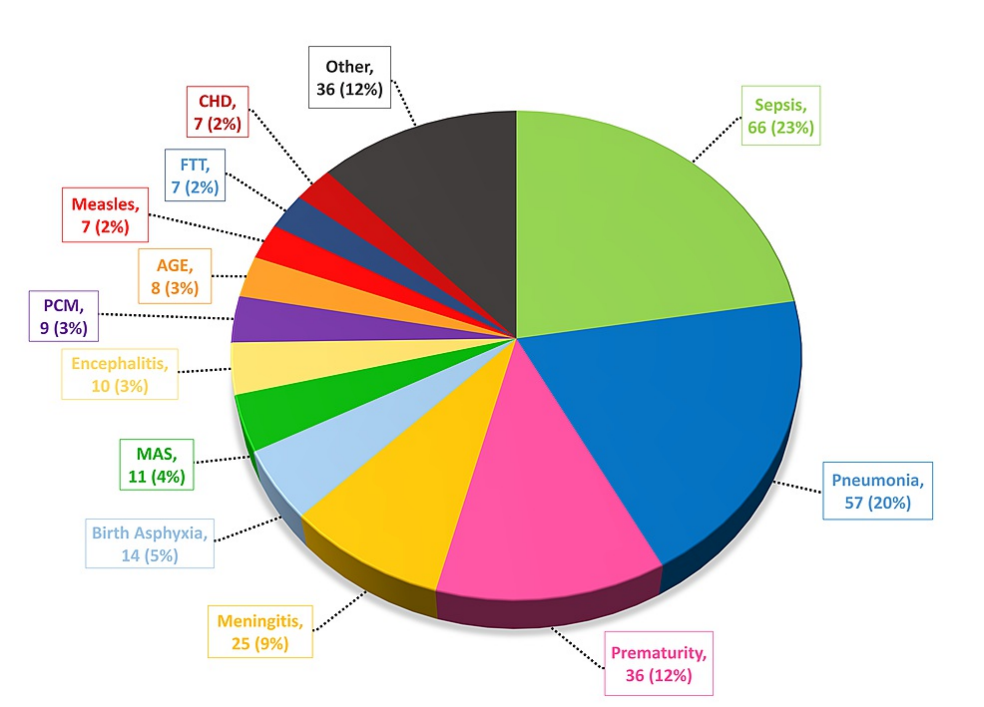
Causes of pediatric mortality CHD: congenital heart disease; PCM: protein-calorie malnourishment; FTT: failure to thrive; AGE: acute gastroenteritis; MAS: meconium aspiration syndrome

According to our study, the leading causes of mortality were sepsis (66 deaths; 23%), pneumonia (57 deaths; 20%), prematurity (36 death; 12%), meningitis (25 deaths; 9%), and birth asphyxia (14 deaths; 5%). Other causes include meconium aspiration syndrome, encephalitis, protein-calorie malnutrition, acute gastroenteritis, measles, failure to thrive, and congenital heart disease, stated in decreasing order of the number of admissions (Figure 1). The most common cause of pediatric morbidity was pneumonia (1115 cases), followed by meningitis (544 cases), acute gastroenteritis (369 cases), enteric fever (262 cases), sepsis (232 cases), measles (212 cases), febrile fits (208 cases), malaria (206 cases), protein-calorie malnutrition (167 cases), dengue (76 cases), and failure to thrive (51 cases).

A vital part of our study was to examine various disease trends over time. We selected diseases that had the highest morbidity and mortality rates at the tertiary care center and presented them in a graphical form to see whether they follow a seasonal/cyclical pattern. The number of admissions for each disease was entered four times a year (March, June, September, and December) to avoid confusion when deciphering trends for the five-year study. Where deemed appropriate, a trend line (linear or polynomial) was added.

**Figure 2:**
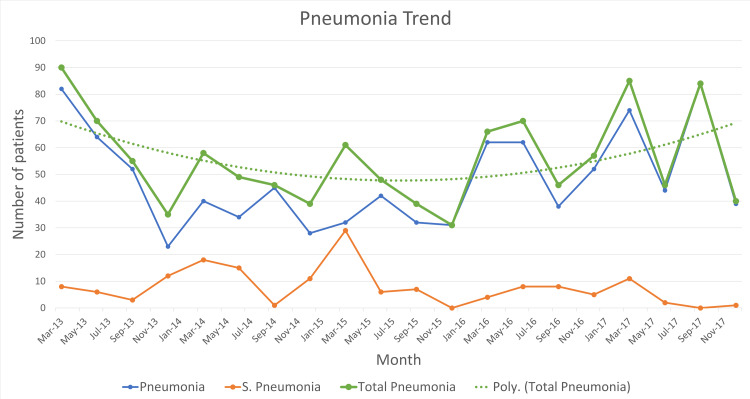
Pneumonia trend

According to the Integrated Management of Childhood Illnesses (IMCI), pneumonia was reported as either a case of pneumonia or severe pneumonia. When presented graphically, the total number of admission for pneumonia (severe and non-severe cases) was also shown. It can be seen in Figure 2 that the highest number of patients admitted diagnosed with pneumonia occurred in March 2013 (a total of 90 patients), whereas the lowest number occurred in December 2015 (31 patients). In March, pneumonia constantly exhibits its peak annually, while in December, pneumonia admission appears to drop (except in 2016 wherein an unexpected rise is seen during December). Peaks in June 2016 and September 2017 do not follow the cyclical pattern of pneumonia seen in previous years. A polynomial trend line shows that pneumonia admissions were decreasing (probably as an effect of the introduction of the pneumococcal vaccine in the EPI schedule), up until 2016, wherein an unanticipated rise was seen.

**Figure 3:**
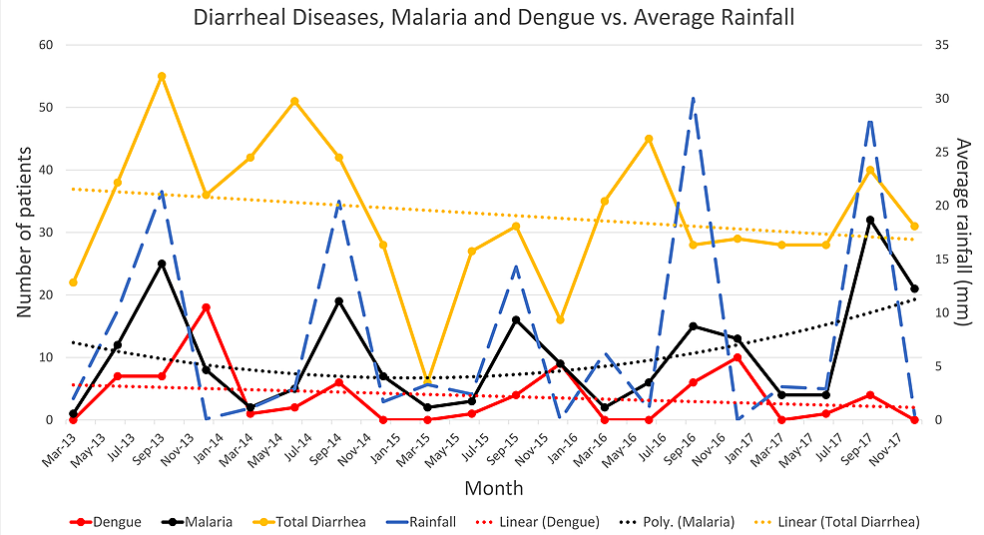
Diarrheal diseases, malaria, and dengue trends alongside average rainfall (mm)

Some diseases were noted to be showing seasonal trends, particularly the rainy season in Karachi, and were, hence, plotted alongside average rainfall (in mm) to show how the trends varied according to the amount of precipitation occurring in the region. As seen in Figure 3, average rainfall shows peaks in September every year (August being the month where Karachi receives the highest amount of rainfall). It can be observed that diarrheal diseases, including acute gastroenteritis and enteric fever, show three peaks that coincide with the average rainfall peak (September 2013, 2014, and 2015). In 2014 and 2016, however, the peak of diarrheal diseases precedes the peak of average rainfall and occurs in June, but overall, a linear trend line shows that the diarrheal diseases are on a decreasing trend with the highest admissions in September 2013 (55 cases) and the lowest admissions in March 2015 (six cases).

Similarly, malaria and dengue were also associated with precipitation. Malaria consistently showed peaks in September that coincide with average rainfall peaks throughout the five-year study period. On the other hand, dengue showed peaks during the rainy season in September 2014 and 2017 only, and other peaks were found in the dry periods (in December 2013, 2015, and 2016). Trends in malaria show that admissions were decreasing initially but are now on an increase, with the highest number of admissions occurring in September 2017 (32 cases) and the lowest in March 2013 (one case). However, a linear trend line through dengue shows that admissions are decreasing, with the highest number of cases annually in 2013 (32 cases) and the lowest for 2017 (five cases).

**Figure 4:**
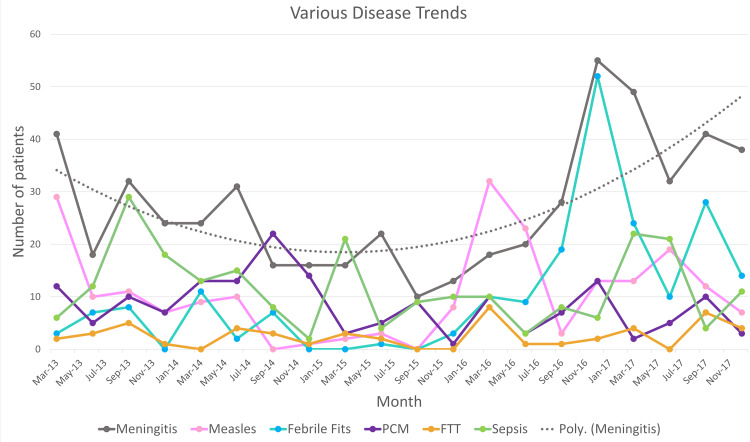
Trends shown by various diseases

Some diseases did not show any cyclical or seasonal trend and they were grouped together and presented in Figure 4. The major cause of morbidity and mortality among these was meningitis, which showed peaks randomly in March and September 2013, June 2014, June 2015, December 2016, and September 2017. The highest number of admissions for meningitis occurred in December 2016 (55 cases) and the lowest in September 2015 (10 cases), while a polynomial trend line shows that, while initially, the number of cases was decreasing, meningitis is on the rise again recently. Other significant diseases include measles which shows outbreaks in March 2013, March 2016, and June 2017.

**Figure 5:**
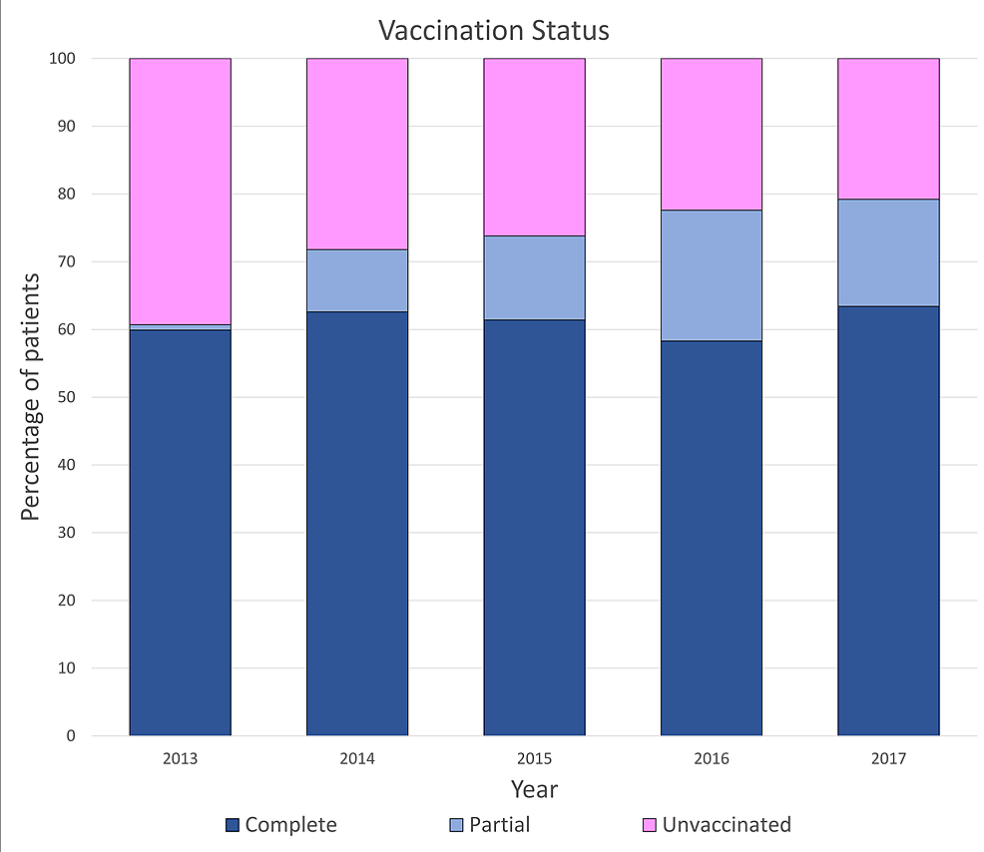
Vaccination status through the years

The vaccination status of the patients was also noted and a total of 1904 (61.2%) children were completely vaccinated according to their age, 341 (11.0%) were partially vaccinated, and 865 (27.8%) were unvaccinated (Figure 5). Over the course of the study, the number of children who received complete vaccination has shown an increasing trend while the sum of unvaccinated children has constantly declined.

## Discussion

In the pediatric world, acute respiratory infections are considered to be the leading cause of morbidity and mortality among children under the age of five years, with most of the burden weighing towards the developing countries where a large number of deaths are attributed to pneumonia. Incidence in the developing countries is estimated to be decreasing, with 0.29 episodes per child-year in 2000 [10], which estimates to about 151 million episodes per year, to 0.22 episodes per child-year in 2010 [11,12].

Trends in the cases of pneumonia over the five-year duration of our study were examined as a part of this report. After the widespread introduction of the conjugate pneumococcal vaccine in EPI in 2012, the number of cases presenting to the tertiary care center seems to fall during the initial years. However, a polynomial trend line through the course of five years (Figure 2) shows that pneumonia had a resurgence in recent years. This can be multi-factorial, as a consequence of antibiotic resistance, improper vaccine administration, nutritional deficiencies, etc.

Antibiotic resistance may be a key factor as many studies have shown that there is a global resurgence of infectious diseases due to resistant bacterial species. This may be more evident in the developing countries like Pakistan, due to the fact that WHO recommends early antibacterial coverage for severe and non-severe cases in low- and middle-income countries (because of high mortality rates), but the predominance of viral etiology of pneumonia raises the question of antibiotic overuse. Randomized controlled trials comparing outcomes of antibiotic and placebo treatments for non-severe pneumonia, conducted in India and Denmark, show that there was no significant difference between the two treatment outcomes [13,14]. Comparatively, one similar study conducted in Pakistan also showed that there was no significant difference between the two [15].

Yearly trends in pneumonia show that, in our study period and setting, February to March seems to be a favorable time for pneumonia admissions, showing peaks in this period every year. This is supported by the findings in a study conducted in Karachi, Pakistan by Raza et al. in 2012 which showed that incidence of community-acquired pneumonia was high in the winter and spring season (i.e., mid-December to mid-April) [16]. However, they have provided only two years of data, while we have statistics for five year. A study conducted in the Netherlands also shows that the highest number of pneumonia admissions occur during the winters, but the weather conditions are very different from Karachi, Pakistan [17].

Meningitis was the fourth highest cause of mortality and the second highest cause of morbidity in our study. While examining the trends for meningitis, we can see that the number of admissions per year showed an overall decrease. However, between September 2016 and April 2017, there was an unprecedented rise of meningitis admissions to the tertiary care hospital which might indicate a local outbreak. The case fatality rate for meningitis during the five-year study period was 4.6% (25 deaths among the 544 cases). This is in contrast to the case fatality rates presented in two Japanese studies conducted by Sunakawa et al. and Shinjoh et al., which showed a decreasing mortality rate of 3.0% and 2.0%, respectively, whereas in our case it was observed to be increasing [18,19]. Another study conducted in Taiwan shows decreasing mortality but increasing morbidity over a period of 29 years [20]. On the other hand, our five-year study shows us increasing trends in morbidity and mortality of meningitis.

When studying diarrheal disease trends, we found that the peaks of admission for diarrheal diseases seem to coincide with the peaks of average rainfall in the region, rainfall season in Karachi, Pakistan is approximately around June to September with the highest amount of rainfall occurring in August (Figure 3). However, in the period where there is little or no rainfall (October to May), a high number of admissions for diarrheal diseases was still observed each year. A study conducted by Anyorikeya et al. in Ghana shows similar findings wherein peaks of rainfall were followed by peaks in diarrhea admissions [21]. In addition to which another study carried out locally by Luby et al. shows the highest rate of admission during August, wherein Karachi receives the maximum amount of rainfall [22]. Furthermore, the high rate of admissions during dry periods can be explained by another study carried by Patel et al., which studied the global seasonality of rotavirus infections and stated that rotavirus infection rates are high during winter in temperate regions like Pakistan [23].

We had a total of 396 cases of acute gastroenteritis and 262 cases of enteric fever during the course of the five-year study period, making them the third and fourth most common cause of morbidity presenting to the tertiary care hospital, respectively. During the period of our study, the rotavirus vaccine had not yet been introduced in the EPI schedule (it was introduced in 2018). Regardless, a linear trend line through the five-year period shows a decrease in the number of admissions.

In the case of malaria, we noticed that during the study period, malaria consistently showed peaks in September. As discussed previously, the month of August is when Karachi faces the highest amount of precipitation. The female Anopheles mosquito, which breeds in fresh water and is the vector responsible for spreading malaria, has a suitable environment to multiply rapidly during this period and, hence, high precipitation is followed by high admission rates of malaria making the two values directly proportional [24]. The relationship between malaria and precipitation has been studied in extensive detail in a 10-year study by Gunda et al. conducted in Zimbabwe, which shows that malaria incidence is significantly related to precipitation with a one-month lag period, as shown in our study as well, with peak rainfall occurring in August and peak hospital admissions for malaria occurring in September [25].

A decreasing trend was observed in malaria over the initial four years, but it had the highest number of admissions among all years in September 2017, which might indicate an outbreak.

Dengue, like malaria, has shown seasonal variations according to various studies and in tropical countries like Latin America and Southeast Asia, where dengue is endemic, has been positively associated with rainfall [26]. A study conducted by Mutheneni et al. in India, which also receives monsoon rains in the same period as Pakistan, shows a moderate to a strong positive correlation between total precipitation and dengue cases [27]. However, in our study, dengue trends failed to show any consistency as only some peaks, occurring in September, seem to be associated with rainfall (2014 and 2017) while others occur during the dry period in December. Overall, a linear trend line over the course of five years shows that dengue has presented a decreasing trend in our study showing that dengue prevention and awareness campaigns are showing a slow, but steady effect.

The EPI was initiated in Pakistan in 1978 covering multiple diseases, including childhood tuberculosis, poliomyelitis, diphtheria, pertussis, tetanus, and measles. More vaccines have been added since then, namely the hepatitis B vaccine (in 2002), the pentavalent vaccine (in 2009), the pneumococcal vaccine (PCV10) (in 2012), the inactivated polio vaccine (in 2015), and most recently, the rotavirus vaccine (in 2018) [28].

When it comes to vaccination coverage in Pakistan, research is highly valuable due to the lack of extensive data in this regard. WHO states that vaccination coverage in 2014-2015, as recorded by Pakistan Social and Living Standards Measurement (PSLM), was 88% which seems overestimated given the fact that our study, conducted in a large metropolitan city such as Karachi, exhibited a very low vaccine coverage (only 61.2% were completely vaccinated) [28]. This has also been reflected in a multicenter study conducted in Karachi by Siddiqui et al., which states that only 51.8% of children were completely vaccinated [29]. Comparatively, in a study carried out by Hoest et al., vaccination coverage in Naushero Feroze, a rural city in Pakistan, was 88.3% which shows that there is an alarmingly high regional variation that needs to be addressed [30]. In our tertiary care center in Karachi, Pakistan, we observed that a high percentage of children had incomplete immunization, although, the majority had a bacillus Calmette-Guerin (BCG) scar. However, due to the lack of immunization card with most parents presenting to the hospital, authentic data regarding immunization status could not be obtained.

As mentioned above, our study was limited by the questionable authenticity of vaccination status due to the lack of a proper validation system. Moreover, being a single-center study, the results can be representative of the local population but cannot be applicable country-wide. A widespread, multi-center study needs to be carried out in order to grasp the full extent of the problem.

## Conclusions

High rates of morbidity and mortality in Pakistan, as compared to the rest of the developing world, make it extremely important to find out the causes and eliminate the contributing factors as a radical reduction would affect global mortality rates and help WHO reach its goals of global well-being.

Our viewpoint highlights the main precipitating elements causing the increase in morbidity and mortality, which most importantly include low vaccination coverage in the low socioeconomic class in the urban region of Karachi as well as the cyclical/seasonal patterns shown by some diseases. Research should be focused on major diseases which are resurging (including pneumonia, malaria, and meningitis) and their causative factors because they are creating a huge impact on morbidity and mortality. This data can help us predict future admission rates and prepare accordingly and aid policymakers to determine who is to blame and above all, how to rectify the hindrance of goal achievement with regards to child mortality.
